# Scorpions from Mexico: From Species Diversity to Venom Complexity

**DOI:** 10.3390/toxins8010002

**Published:** 2015-12-24

**Authors:** Carlos E. Santibáñez-López, Oscar F. Francke, Carolina Ureta, Lourival D. Possani

**Affiliations:** 1 Departamento de Medicina Molecular y Bioprocesos, Instituto de Biotecnología, Universidad Nacional Autónoma de México, Avenida Universidad 2001, Apartado Postal 510-3, Cuernavaca Morelos 62210, Mexico; possani@ibt.unam.mx; 2 Colección Nacional de Arácnidos, Instituto de Biología, Universidad Nacional Autónoma de México, Circuito exterior s/n, Ciudad Universitaria, Copilco, Coyoacán A.P. 70-233, Distrito Federal 04510, Mexico; offb@ib.unam.mx; 3 Laboratorio de Genética Molecular, Desarrollo y Evolución de Plantas, Departamento de Ecología Funcional, Instituto de Ecología, Universidad Autónoma de México, Apartado Postal 70-275, Ciudad Universitaria, Distrito Federal 04510, Mexico; carolina_ureta@hotmail.com

**Keywords:** mexico, neotropical, nearctic, buthidae, diplocentridae, vaejovidae, venom, diversity hotspots

## Abstract

Scorpions are among the oldest terrestrial arthropods, which are distributed worldwide, except for Antarctica and some Pacific islands. Scorpion envenomation represents a public health problem in several parts of the world. Mexico harbors the highest diversity of scorpions in the world, including some of the world’s medically important scorpion species. The systematics and diversity of Mexican scorpion fauna has not been revised in the past decade; and due to recent and exhaustive collection efforts as part of different ongoing major revisionary systematic projects, our understanding of this diversity has changed compared with previous assessments. Given the presence of several medically important scorpion species, the study of their venom in the country is also important. In the present contribution, the diversity of scorpion species in Mexico is revised and updated based on several new systematic contributions; 281 different species are recorded. Commentaries on recent venomic, ecological and behavioral studies of Mexican scorpions are also provided. A list containing the most important peptides identified from 16 different species is included. A graphical representation of the different types of components found in these venoms is also revised. A map with hotspots showing the current knowledge on scorpion distribution and areas explored in Mexico is also provided.

## 1. Introduction

The order Scorpiones is a distinctive group of arachnids including the oldest fossils in the class, dating back to almost 433–438 mya [1,2]. Scorpion groundplan has remained almost unchanged ever since they became fully terrestrial. Despite this conservative morphology, scorpions successfully colonize different ecosystems, from the deepest caves in Mexico (e.g., *Alacran tartarus* Francke, 1982 at 900 m below ground level [3]) to some of the highest peaks in the Andes Cordillera (*Orobothriurus huascaran* at 4910 m [4]), and some harsh environments such as dunes or deserts with high temperatures [5,6]. Scorpions are distributed worldwide except in Antarctica, the boreal areas and some oceanic islands [7]. The greatest diversity of scorpions occurs in subtropical areas and decreases towards the equator and the poles [6].

Although they are not as morphologically diverse as opilionids, spiders and mites, the order comprises nearly 2231 species in 208 genera and 20 families [8,9], including the species enlisted in this contribution.

Scorpion systematics is problematic and in continual change. Prendini and Wheeler [10] proved that a more detailed study on scorpion phylogenetics is necessary to achieve uniformity in the taxonomy and a stable classification. Therefore, several taxonomic revisions on different scorpions’ genera are more common these days and the number of species has increased rapidly, along with changes in their classification. However, recent phylogenetic and phylogenomic approaches to reveal species evolutionary history are contrasting with the morphological phylogenetic indications [11].

The remarkable scorpion diversity of Mexico has been recognized for more than 30 years. Several authors [12,13,14,15,16,17,18,19] have summarized the diversity of scorpions in Mexico (for previous works before 1970 of Mexican scorpions refer to [16,19]).

Scorpion diversity in Mexico, with 281 species described (until October 2015), represents more than 12% of the worldwide diversity (Table 1). Although recent efforts have been made to collect scorpions across the entire country (*i.e.*, Revisionary Systematics of the family Vaejovidae Thorell, 1876 project), there are several areas not yet accessed or that remain poorly sampled (e.g., Sinaloa, Sonora, Tamaulipas, and Coahuila). Even though most of the species are recorded in several localities and from several specimens, many others are recorded only from type localities, and from single specimens, which could affect the endemism levels reported [16,19]. When the sampling is not exhaustive, the information available might be deficient. In addition, for the same reason, the species considered endemic of one given place can eventually be found in other areas.

**Table 1:** Comparative list between Mexican scorpion fauna and world’s diversity. The list includes known introduced species, but not subspecies. Percentages are of the total genera and species worldwide represented in the country.

Families	Worldwide		Mexico			
	Genera	Species	Genera	Species	% Genera	% Species
Akravidae	1	1	0	0	0	0
Bothriuridae	16	150	0	0	0	0
Buthidae	92	1054	2	44	2	4
Caraboctonidae	5	31	3	9	60	29
Chactidae	12	178	1	1	8	1
Chaerilidae	1	40	0	0	0	0
Diplocentridae	10	121	3	58	30	48
Euscorpiidae	4	51	3	8	75	16
Hemiscorpiidae	1	15	0	0	0	0
Heteroscorpionidae	1	6	0	0	0	0
Hormuridae	11	81	0	0	0	0
Iuridae	4	39	0	0	0	0
Pseudochactidae	3	6	0	0	0	0
Scorpionidae	9	152	0	0	0	0
Scorpiopidae	6	67	0	0	0	0
Superstitioniidae	1	1	1	1	100	100
Troglotayosicidae	2	5	0	0	0	0
Typhlochactidae	4	11	4	11	100	100
Urodacidae	2	22	0	0	0	0
Vaejovidae	23	201	21	149	91	74
Totals	208	2231	38	281	18	13

In the present contribution, the Mexican scorpion species diversity is revised. It is not the scope of this contribution to cover the entire history of scorpion taxonomy research in Mexico, but to briefly update it from the last contribution in 2005. To learn about the history of scorpion studies in Mexico before the year 2005, please refer to Lourenco and Sissom [16] and Sissom and Hendrixson [19]. Updated species lists and their distribution within Mexican political divisions are provided. Although several authors argue against the study of distributional and endemism patterns using geopolitical boundaries, we agree with other authors (*i.e.*, [19]) that these studies are necessary to provide enough information for governmental decisions on biodiversity conservation. A review on the knowledge of general aspects of scorpions, such as ecology, behavior and venomic studies for Mexican scorpion species, is also included.

### Scorpion Taxonomy and Systematics

As mentioned above, scorpion systematics and taxonomy have been changing in recent years. We agree with Prendini and Wheeler [10] on the need for a rigorous phylogenetic analysis using as much information as possible in order to propose a valid classification. Therefore, for this contribution, we follow Prendini’s [8] familial and generic classifications, but with some modifications. These modifications are based on the observations recently discussed elsewhere (*i.e.*, [20,21,22]).

We recognize, therefore, 2231 species in 208 genera and 20 families (see Table 1 and [8]).

## 2. Mexican Scorpion Diversity

**Figure 1:**
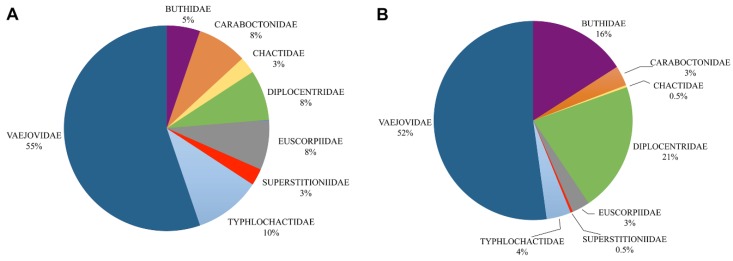
Proportion of scorpion diversity in Mexico. (**A**) Percentage of genera in the families of Mexican scorpions; (**B**) Percentage of species in the families of Mexican scorpions.

**Figure 2:**
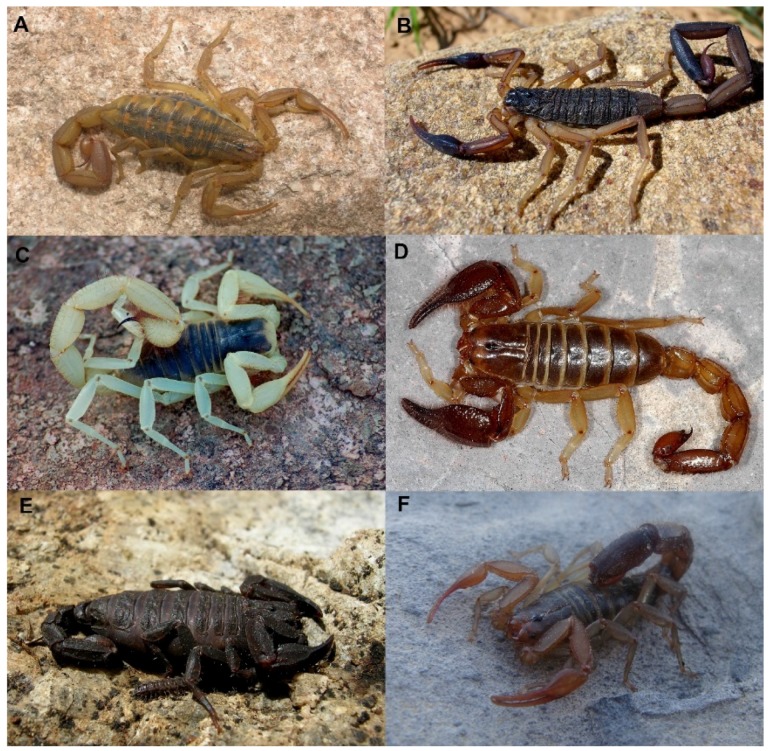
Representative scorpion species of families from Mexico: (**A**) Adult ♀ *Centruroides limpidus* (Karsch, 1879) (Buthidae); (**B**) Adult ♀ *Centruroides nigrimanus* (Pocock, 1898) (Buthidae); (**C**) Adult ♂ *Hadrurus obscurus* Williams, 1970 (Caraboctonidae); (**D**) Adult ♂ *Diplocentrus colwelli* Sissom, 1986 (Diplocentridae); (**E**) Adult ♀ *Megacormus segmentatus* (Pocock, 1900) (Euscorpiidae); (**F**) Adult ♂ *Franckeus kochi* (Sissom, 1991) (Vaejovidae).

**Figure 3:**
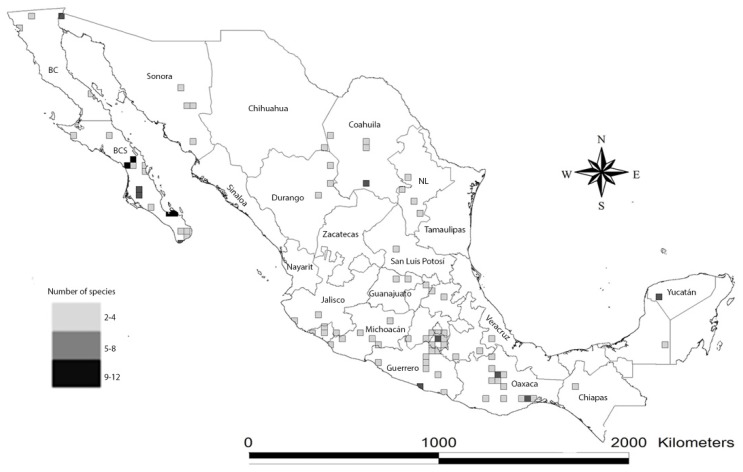
Hotspots of scorpion species richness in Mexico, expressed as the number of species per quarter-degree square mapped onto a Mexican geopolitical division map.

We recognize eight families, 38 genera and 281 species distributed in Mexico (Figure 1, Figure 2 and Figure 3; Table 1; Supplementary Materials). Vaejovidae Thorell, 1876 comprises 52% of the Mexican scorpion species diversity, Diplocentridae Karsch, 1880 and Buthidae Koch, 1837 represent 21% and 16% respectively, and lower diversity is found in families Typhlochactidae Mitchell, 1971, Caraboctonidae Kraepelin, 1905 and Euscorpiidae Laurie, 1896, representing 4%, 3% and 3%, respectively; and the rest of the families the remaining 1% (Chactidae Pocock, 1893 and Superstitioniidae Stahnke, 1940; see Figure 1).

Of the eight families in Mexico, only Typhlochactidae is strictly endemic to the country (see section 2.7, although Superstitioniidae is 100% represented in Mexico. Vaejovidae is distributed in Mexico and US; however, it is by far more diverse in Mexico than in US. Twenty-one genera are recorded in Mexico (eight endemic) while 13 are found in US (three endemic). Diplocentridae is also distributed in Mexico and US, but its distribution extends to northern South America and the Caribbean islands. Two genera are endemic to Mexico. Three of the four genera comprised in Euscorpiidae are found in Mexico, two genera are endemic. The only chactid representative in Mexico is endemic, and Superstitioniidae and Caraboctonidae are shared between Mexico and US, except for one caraboctonid genus endemic to central Mexico (see section 2.2). Finally, one buthid genus is endemic to the Pacific coast of Mexico.

### 2.1. Family Buthidae C.L. Koch, 1837

Two genera are represented by nearly 44 extant species, plus two fossil species of a third genus (*Tityus*) from Chiapas [23] (not considered in our counts). Genus *Centruroides* Marx, 1860 is by far the most diverse scorpion genus of this family in Mexico (Figure 2A,B). Currently, this genus comprises 42 species distributed acrosss almost the entire territory, but its highest diversity is found in Southern Mexico (in the states of Michoacán, Guerrero and Oaxaca). However, this estimation is still far from reality and it will be outdated soon. Despite the great interest in the study of the diversity of this genus in Mexico by several authors (*i.e.*, [17,24,25,26]), the taxonomy of this genus has been long related to coloration patterns and morphometrics, making the diagnosis of some species difficult. Also, the vast majority of studies on *Centruroides* are mostly of its venom, given that less than a dozen Mexican species have highly toxic venoms to humans (see venomic studies section).

Recently, Francke *et al.* [27] described a new genus from the Pacific coast of Guerrero and Oaxaca (Southern Mexico). *Chaneke* Francke, Teruel & Santibáñez-López, 2014 is a small orange-reddish brown buthid, with decreasing neobothriotaxy, and both species are morphologically similar to some species of *Alayotityus* Armas, 1973 from Cuba. This genus is poorly sampled, poorly represented in museum collections and its venom, ecology and behavior have not been studied at all.

### 2.2. Family Caraboctonidae Krapelin, 1905

Three genera are distributed in Mexico including the biggest scorpions in North America (reaching up to 150 mm). Although some *Centruroides* and some diplocentrids reach this length, *Hadrurus* and *Hoffmannihadrurus* species have a more robust body. *Hadrurus* Thorell, 1876 (Figure 2C) is represented by six species, all of them distributed in Northern Mexico. Six species are recorded in Baja California Peninsula and one in Sonora. *Hoffmannihadrurus* Soleglad & Fet, 2004 is regarded as distinct from *Hadrurus* Thorell, 1876 based on several characters. Two species are included in this genus: *Hoffmannihadrurus aztecus* (Pocock, 1900) distributed in Cuicatlán-Tehuacán valley in Oaxaca and Puebla; and *Hoffmannihadrurus gertschi* (Soleglad, 1976) distributed in southern Morelos and northern Guerrero. Finally, one species of *Anuroctonus* is distributed in Baja California (*Anuroctonus pococki* Soleglad and Fet, 2004).

### 2.3. Family Chactidae Pocock, 1893

*Nullibrotheas allenii* (Wood, 1863) is an endemic member of the Mexican fauna and the only representative of family Chactidae in the Nearctic region. It is distributed in the southern half of Baja California Sur [5]. It is easily recognized by the presence of six trichobothria in the ventral surface of the pedipalp patella. This species is light to pale yellow brownish and medium sized.

### 2.4. Family Diplocentridae Karsch, 1880

Scorpions of this family are easily recognized by the presence of a subaculear tubercle, also by the pedipalp chela manus mostly rounded and robust, and because most of the species are fossorial, living in burrows on the ground, the pelophilous ecomorphotype sensu [28].

*Bioculus* Stahnke, 1968 is endemic to Mexico and it comprises five species. It is distributed in Baja California Sur (four species), and one species can be found in Guerrero. Its disjunctive distribution pattern most likely reflects the separation of the Baja California Peninsula from mainland Mexico almost 5 mya [29,30]; however, a phylogenetic analysis and a molecular dating of the divergence of this genus are missing.

*Diplocentrus* Peters, 1861 is the most diverse genus of family Diplocentridae. It is distributed from southern US to Honduras [21] (Figure 2D). Mexico harbors almost 84% percent of the species of the genus. Scorpions of this genus are present in almost all Mexican territories except in the Baja California Peninsula, where they are replaced by *Bioculus*. Species of *Diplocentrus* size ranges from the smallest *Diplocentrus bereai* Armas and Martín-Frías, 2004 (around 23 mm) to the biggest *Diplocentrus taibeli* (Caporiacco, 1938) reaching almost 120 mm.

*Kolotl* Santibáñez-López, Francke and Prendini, 2014 was recently created to accommodate two species with increasing neobothriotaxic pedipalps. It is distributed on the coast of Guerrero and in central Michoacán [31]. These scorpions are also big with a robust body (but not as big as *Hadrurus* species) living in crevices (the lithophilous ecomorphotype sensu [28]).

### 2.5. Family Euscorpiidae Laurie, 1896

Three of the four genera of this family are distributed in Mexico. Two of them (*Megacormus* Karsch, 1881 and *Troglocormus* Francke, 1981) are endemic to the Sierra Madre Oriental, from Tamaulipas to Oaxaca, whereas *Plesiochactas* Pocock, 1900 is also distributed in Chiapas and Guatemala. Scorpions of *Megacormus* and *Plesiochactas* are colored black or dark brown, while one species of *Troglocormus* is unpigmented and the other one is dark. Their size ranges 35–65 mm. They have been neglected in the past because they are sometimes hard to find, and also because they do not reflect UV light as brightly as the other scorpion families.

*Megacormus* Karsch, 1880 is represented by four corticolous species [28] (Figure 2E) distributed from Tamaulipas to Oaxaca and Veracruz. 

*Plesiochactas* Pocock, 1900 is represented by two species, one in Oaxaca and Veracruz and the other one is in Chiapas, but it is also present in Guatemala. *Troglocormus* Francke, 1981 is represented by two troglobitic species distributed in caves of San Luis Potosí and Tamaulipas, respectively.

### 2.6. Family Superstitioniidae Stahnke, 1940

This family is monotypic and endemic to southern Arizona and to the Baja California Peninsula. *Superstitionia donensis* Stanhke, 1940 is a small scorpion (approx. 25–30 mm long), with a shiny and lustrous yellow to orange-brown body coloration with irregular dusking black marks. This species is widely distributed in the Baja California Peninsula, and it inhabits different ecosystems: from deserts to semi deserts and rocky hills.

### 2.7. Family Typhlochactidae Mitchell, 1971

Scorpions of this family are strictly troglomorphic, and although only few species have been collected in epigean habitats (e.g., *Typhlochactas sissomi* Francke, Vignoli & Prendini, 2009), most of the species are strictly troglobitic. This family is divided into two subfamilies: Alacraninae Vignoli and Prendini, 2009; and Typhlochactinae Mitchell, 1971. Subfamily Alacraninae comprises one genus *Alacran* Francke, 1982 and three species (see Supplementary Materials). These scorpions live in some of the deepest caves in the world (*ca.* 900 m below surface). They are well adapted to the environment. Some specimens of *A. tartarus* Francke, 1982 and *A. triquimera* Santibáñez-López, Francke and Prendini, 2014 have been collected or observed under water (Mr. William Steele, pers. observations).

Subfamily Typhlochactinae comprises three genera: the monotypic genera *Sotanochactas* Francke, 1986 and *Stygochactas* Vignoli and Prendini, 2009; and the polytypic genus *Typhlochactas* Mitchell, 1971 (with six species). Scorpions of this family are distributed and mostly restricted to caves of the Sierra Madre Oriental, from Tamaulipas to Oaxaca.

### 2.8. Family Vaejovidae Thorell, 1876

This family is the most diverse in Mexico. It is distributed from Canada to Guatemala [32]. Scorpions of this family are a major Nearctic component of the Mexican fauna. Most of the species inhabit desert, semi-desert or dunes and they are highly specialized. Mexico harbors currently 149 species in 21 genera (eight genera are endemic). Higher level systematics (*i.e.*, subfamily level) and classification is currently in dispute. For the purposes of this contribution, we recognize three subfamilies: Syntropinae Kraepelin, 1905 (recently reviewed in [22,33]); Smeringurinae Soleglad and Fet, 2008 (although its monophyly has not been tested) and Vaejovinae Thorell, 1876.

Subfamily Syntropinae comprises 11 genera and 56 species; however, several major generic revisions are underway and this diversity will increase. Genus *Balsateres* González-Santillán and Prendini, 2013 is monotypic and it is endemic to central Mexico (Michoacán and Mexico). Genus *Chihuahuanus* González-Santillán and Prendini, 2013 is represented by eight species, all of them distributed in northern Mexico and shared along the border with US. Genus *Kochius* Soleglad and Fet, 2008 is represented by nine species, mostly distributed in the Baja California Peninsula, except for *Kochius sonorae* (Williams, 1971), which is distributed in Sonora. Genus *Konetontli* González-Santillán and Prendini, 2013 (endemic to Mexico) is represented by 10 species distributed along the Pacific coast in the states of Guerrero, Michoacán, Jalisco, Nayarit and one species in Baja California Sur (which lives in pine oak forest; [34]). Genus *Maaykuyak* González-Santillán and Prendini, 2013 is represented by two species: one restricted to Baja California Sur [*Maaykuyak vittatus* (Williams, 1970)], and the other one [*Maaykuyak waueri* (Gertsch and Soleglad, 1972)] is distributed in northern Mexico (Durango, Chihuahua, Coahuila, and Nuevo Leon) and the US (Texas). Genus *Mesomexovis* González-Santillán and Prendini, 2013 (endemic to Mexico) is represented by seven species; they are distributed in Central and Southern Mexico. Genus *Paravaejovis* Williams, 1980 is represented by 11 species, all distributed in northern Mexico and Baja California Peninsula. Genus *Syntropis* Kraepelin, 1900 is represented by three species found in the Baja California Peninsula; these scorpions are among the largest in the vaejovids, and they are lithopilous (Prendini, 2001). Genus *Thorellius* Soleglad and Fet, 2008 is represented by three endemic species to Central Mexico (Colima, Jalisco, Michoacán, and México); they also are among the biggest vaejovids in Mexico. Genus *Vizcaino* González-Santillán and Prendini, 2013 is monotypic and endemic to the Vizcaino desert in the Baja California Peninsula; and the genus *Kuarapu* Francke and Ponce, 2011, also monotypic and endemic to the Balsas Depression in Michoacán.

Although the monophyly of subfamily Smeringurinae has not been tested in a phylogenetic context [22], the following genera are considered within the subfamily: *Paruroctonus* Werner, 1934 is represented in the country by 15 species (half its total diversity), which are distributed in northern Mexico (Sonora, Coahuila, Chihuahua) and Baja California; however, *Paruroctonus gracilior* (Hoffmann, 1931) is widely distributed in Texas, New Mexico and Arizona in US, and in Coahuila south to Aguascalientes and San Luis Potosí in Mexico. These scorpions are psammophilous [28]. *Smeringurus* Haradon, 1983 is represented by two species in Baja California, both psammophilous [28]; and *Vejovoidus* Stahnke, 1974, monotypic, endemic to the Vizcaino desert and strictly psammophilous.

Also, the monophyly of the subfamily Vaejovinae has not been tested; however, the following genera are currently considered within this subfamily: *Franckeus* Soleglad and Fet, 2005 (previously known as the “*nitidulus*” group within *Vaejovis*; Figure 2F) is represented by six species endemic to Mexico, distributed in Central Mexico, Northern Mexico and with one species in Baja California Sur. Scorpions of this genus are lithophilous [28]. *Pseudouroctonus* Stahnke, 1974 is represented by nine species distributed in northern Mexico and the Baja California Peninsula. Some species of this genus are troglobites and inhabit caves in Coahuila. *Uroctonites* Williams and Savary, 1991 with only one species in Sonora shared with Arizona, US. And finally, *Vaejovis* Koch, 1836, which is the most diverse genus of this family in Mexico with 40 species distributed from the north to the south (except in the Yucatán Peninsula) of Mexico. Most of the species are lapidicolous and corticolous; however, *Vaejovis gracilis* Gertsch and Soleglad, 1972 is a troglobite inhabiting caves in Veracruz.

There are three genera with *insertae sedis* status in the family Vaejovidae: *Gertschius* Graham and Soleglad, 2007, includes two species distributed in Sonora; *Serradigitus* Stahnke, 1974, which is represented by 14 species, most of them distributed in the Baja California Peninsula and the rest in northern Mexico (Sonora, Coahuila), and most of the species of this genus is lithophilous; and *Stahnkeus* Soleglad and Fet, 2006 represented by four species in Sonora and Baja California.

## 3. Geographical Hotspots and Environmental Variables as Indicators of Explored Areas in Mexico

In order to know the extent of our knowledge on scorpion distribution in Mexico, the status of exploration in the country, and to serve as a base for study on the endemic hotspot distribution maps, we created a map plotting hotspots using a Mexican map divided into 25 km^2^ cells (quarter of degree grid cells) to show potential areas with more than two species (Figure 3). We calculated the number of species present in each cell. Species’ occurrences were obtained from the records of the CNAN (*Colección Nacional de Arácnidos, Instituto de Biología*, UNAM), and from the literature. We decided not to include the cells where only one species was registered (*i.e.*, type localities of the species with only one record, such as *Typhlochactas sissomi*), because it is known that there are several localities with at least one known scorpion species present in many areas of Mexico. Also, we eliminated dubious records (e.g., records with unknown georeference data or wrong locality name).

The hotspots map shows that (Figure 3) some areas appear to be better sampled (for example, Morelos, in central Mexico) while others are poorly sampled (e.g., Sonora, Chihuahua, Coahuila in northern Mexico). However, there is at least one species recorded in all Mexican states (not shown in the map). Hotspots mapped in Baja California Sur (Baja California Peninsula), the coast of Guerrero, the Isthmus of Tehuantepec, Central Valley in Oaxaca, the Valley of Cuicatlán-Tehuacan (Southern Mexico) and in the Yucatán Peninsula (Eastern Mexico) are consistent with published and unpublished records of several species. The hotspots in Baja California Sur, the Yucatan Peninsula and Central Valley in Oaxaca were previously recognized elsewhere ([5,13,14], respectively). Hotspots in the coast of Guerrero, the Isthmus of Tehuantepec and the Valley of Cuicatlán-Tehuacan were represented by five, six and seven species, respectively, not previously reported.

## 4. Venomic Studies in Mexico

Identification of scorpion venom components (mostly peptides and proteins), their functional characterization, and the cloning of the respective genes are some of the most studied aspects of Mexican scorpions. Over 150 scientific articles have been published since the earliest 1940s. A recent book dealing exclusively with “Scorpion Venom” was recently printed by the editorial Springer [35]. In this section, the most important components found in Mexican scorpions are revised. Scorpions use their venoms for capturing preys or defending themselves from predators. Millions of years of evolution permitted the selection and diversification of specific peptides or proteins with enzymatic activities that normally interfere with the cellular communication among cells of the individuals to which the scorpions inject venom. The peptides identified are exquisite ligands that either block or modify the function of ion-channels. Yet, their function is quite selective. There are peptides that recognize only ion channels from mammalians, crustaceans or insects [36]. These venom components are species specific. Two main peptidic components were found and described: (i) peptides that bind to sodium channels modifying the gating mechanisms [37]; (ii) peptides that recognize potassium channels and block their function [38,39].

Other components that recognize calcium channels were also described [40]. Specific enzymes that act as spreading factors like hyaluronidases are ubiquitous in all scorpion venoms studied thus far [41], but hydrolytic enzymes such as phopholipases or metalloproteinases are also present in these venoms [42,43,44]. None of these components were known, but deadly sting accidents occurred with humans in the country, which motivated the earlier studies conducted by physiologists at the Biomedical Institute of the National Autonomous University of México [45,46].

By the end of the 1970s, a French group under the leadership of Prof. Hervé Rochat published manuscripts describing the isolation and chemical characterization of toxic peptides from the Mexican scorpion *Centruroides suffusus* of Durango [47,48]. Toxin 2 from this scorpion was taken as a prototype of β-scorpion toxins affecting the opening mechanism of Na^+^-channels of excitable cells [49]. The same group of scientists described Toxin II from the North African scorpion *Androctonus australis*, taken as a prototype of the α-scorpion toxins, which modify the closing mechanism of Na^+^-channels [48,50,51,52].

The analyses of the venom components of other scorpions of the species *Centruroides elegans* [53], *Centruroides tecomanus* [54] and mainly *Centruroides noxius* [55,56] were the first biochemical studies conducted in this country with Mexican scorpions. In 1982, Carbone and colleagues described the first scorpion toxin (Noxiustoxin), capable of blocking the function of K^+^-channels [57,58,59]. These findings constitute a landmark discovery that opened up a wide area of research on similar components present in other scorpions around the world, which were important for finally identifying and describing at the three-dimensional level the molecular structure of the first K^+^-channel molecule [60]. Among the Mexican scorpions, *Centruroides noxius* was found to contain the most potent venom against mammals. The LD50 of this venom is 5 µg per 20 g mouse weight [55].

Apart from Noxiustoxin, another very important lethal component was found in this species of scorpion. It was named Cn2, a peptide with 66 amino acid residues very potent and specific ligand for Na^+^-channels of sub-type Nav1.6 [56,61,62,63]. This peptide was taken as a model peptide for preparation of a synthetic vaccine against scorpion venom intoxication [64,65]. It was also used for the development of monoclonal antibodies [61], which served as the basis for the isolation and preparation of single-chain antibodies of human origin, which are now being developed by the group of Dr. Balazar Becerril of the Biotechnology Institute in UNAM [66,67]. Actually, the three-dimensional structure of Cn2 and a protective single-chain antibody were obtained showing the interaction surfaces of toxin and antibody [68].

Other dangerous species of Mexican scorpions studied were *Centruroides sculpturatus* [69,70,71], *Centruroides limpidus* [72,73,74], *Centruroides infamatus* [74]. Advanced studies were conducted with venom components of these scorpions, all belonging to the family Buthidae, genus *Centruroides* [73,74,75,76,77,78,79,80]. The venom from these species is important due to the toxic effect caused by their stings [81].

Novel peptides, specific for K^+^-channels of the *ether-a-go-go* family (ERG-channels), were described and their function studied [82,83]. A series of review articles dealing with the biochemical and molecular biology of these Mexican scorpions is also available in the literature [37,38,83,84,85,86,87,88,89,90].

In addition to the specific peptides that are active on ion-channels of excitable and non-excitable cells, various peptides with different functions were reported, such as: antimicrobial peptides [91,92], anti-malaric peptides [93], anti-convulsive peptide [94], heterodimeric phospholipases [95], metalloprotease activity [44], and immunomodulatory peptides [96,97]. Many new components were isolated and characterized from the venom of scorpions not dangerous to humans [42,43,91,98,99,100]. Among the late described peptides are: Hadrucalcin, extracted from the venom of *Hadrurus gertschi* (= *Hoffmannihadrurus gertschi*), a peptide with affinity to ryanodine receptors and the potential to act as a delivery drug in the treatment of arrhythmogenic disease [40]; Hadrurin also from the venom of *H. gertschi*, a peptide with antimicrobial activity (inhibiting the growth of at least six bacteria) [91]; Phaiodotoxin, a peptide from the venom of the scorpion *Anuroctonus phaiodactylus* (= *Anuroctonus pocoki*, a misidentification), which is lethal to insects but not toxic to human, exhibiting great potential as a bio-insecticidal [43]; and Vm23 and Vm24, from the venom of *Vaejovis mexicanus smithi* (= *Vaejovis smithi*) as an immunosuppressive peptide which inhibits Kv1.3 channels of human lymphocytes, with important pharmacological characteristics suitable for drugs to treat autoimmune diseases [96,97].

Table 2 lists the number of different peptides identified in 16 different species of Mexican scorpions, the most relevant components found and the respective references.

**Table 2:** Number of peptides and the most relevant components isolated from the venom of Mexican scorpions.

Species	# Peptides	Most Relevant	References
*Anuroctonus pococki*	8	Phaiodotoxin	[43]
*Centruroides elegans*	12	CeII8	[101]
*Centruroides exilicauda*	17	Neurotoxin Cex11	[71]
*Centruroides gracilis*	4	Toxin Cg2	[85]
*Centruroides infamatus*	1	Beta toxin Cii1	[74]
*Centruroides limpidus*	20	Cll1, Cll2	[74,79]
*Centruroides margaritatus*	1	Margatoxin	[102]
*Centruroides noxius*	31	Noxiustoxin, Cn2	[56,57,58,59,61,62,63,103]
*Centruroides sculpturatus*	26	CsEv1	[70]
*Centruroides suffusus*	8	CssII	[104]
*Centruroides tecomanus*	37	Clt1	[77,105]
*Hoffmannihadrurus gertschi*	13	Hadrucalcin, Hadrurin, Hge scorpine	[40,91,98,106]
*Mesomexovis punctatus*	15	VpAmp1	[107,108]
*Mesomexovis subcristatus*	8	ViSplp1	[107]
*Thorellius intrepidus*	14	ViCaTx1	[107]
*Vaejovis mexicanus*	22	Vejovine, Vm23, Vm24	[92,96,97]

**Figure 4:**
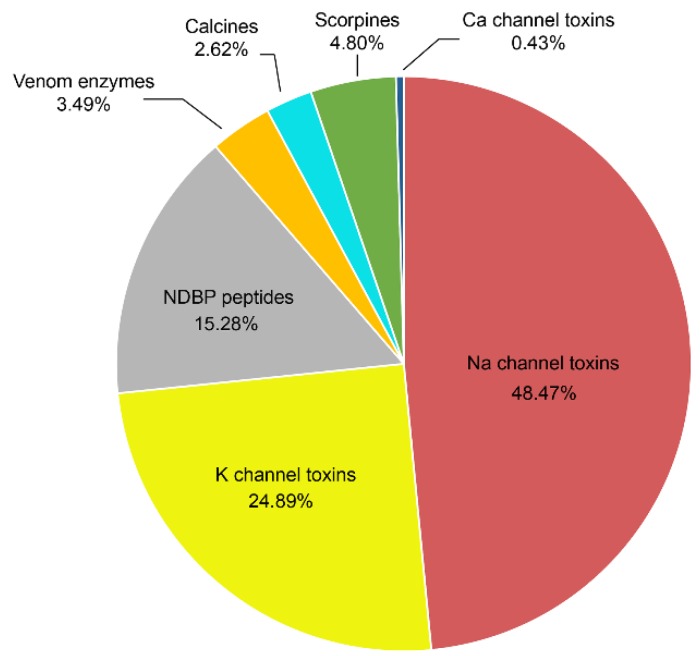
Proportion of the different peptides isolated from the venom of Mexican scorpions.

Figure 4 shows a graphical representation of the distribution of the various important pharmacological components identified in the venom of Mexican scorpions. The most abundant peptides described (over 73%) are those that modify the gating mechanism of Na^+^-channels or block the function of K^+^-channels. This issue is due to the fact that these were the components first isolated from the venoms, because they were responsible for the human intoxication processes. More recently, other components were found such as the non-disulfide containing peptides (NDBP), among which are some with antimicrobial activity. Several enzymes were identified and their function described. Anti-parasitic peptides, like the scorpine-like ones, were isolated and their effect on malaria parasites was reported. Finally, peptides that recognize ryanodine sensitive calcium channels, called calcines, were also isolated and are under study now.

With the advent of the Next Generation Sequencing, the study of venom has changed. Transcriptomic analyses allow us to focus on subsets of target genes (*i.e.*, those coding venom or toxic peptides). Such studies are now more common these days, including the venom gland transcriptomic analyses of *Hoffmannihadrurus gertschi* [106]; *Centruroides tecomanus* [105]; *Mesomexovis punctatus*, *Mesomexovis subcristatus*, *Thorellius intrepidus* and *Vaejovis mexicanus* [107]; and the global transcriptomic analysis of *Centruroides noxius* [103]. However, other studies are in progress and include the first venom gland transcriptomic analysis of one species of *Megacormus*, *Superstitionia donensis* and *Hadrurus concolorous* (Santibáñez-López, in prep.).

## 5. Natural History, Behavior and Ecology Studies of Mexican Scorpions

In sharp contrast with the numerous publications on Mexican scorpions’ taxonomy, systematics and venomics, in the last decade there have been few contributions in these areas on scorpion biology.

In three chapters of the Biology of Scorpions ([6]; Chapters 4–6), many important aspects of life history, behavior and ecology were summarized. However, few species studied or mentioned were distributed in Mexico. Most of the studies were conducted in the Baja California Peninsula and for vaejovids, with the exception of few species in *Centruroides*, and *Megacormus gertschi* Díaz-Najera, 1966. For example, Francke [108] studied the courtship of *Megacormus gertschi*, finding that during mating, the male stings the female, revealing an unusual behavior previously unrecognized in other scorpions.

In the Scorpions 2001 book [109], few aspects on ecology and behavior were covered, but none for Mexican species.

It is not until 2003 when Ponce-Saavedra [110] studied the ecology and distribution of *Centruroides balsasensis*. After that, his laboratory has undertaken several ecological studies on genus *Centruroides* in Michoacán and Guerrero (for example [111]).

Later, Contreras-Garduño *et al.* [112] analyzed the function of the barbed mating plug in *Mesomexovis punctatus* (Karsch, 1879) from Hidalgo. The mating plug is part of the spermatophore produced by the male in order to inseminate the female. In the subfamily Syntropinae (family Vaejovidae), the mating plug has recurved spines that serve to anchor it inside the female’s genital aperture; thus preventing further mating by that female and assuring the paternity of the male that provided the spermatophore. The plug dissolves gradually during the pregnancy and does not interfere with subsequent parturition.

Jiménez-Jiménez and Palacios-Gardell [113] studied the abundance and diversity of scorpions in six oases in Baja California Sur. The richest site contained 14 species and the poorest only three. Abundance at each locality varied with geomorphology, geographic conditions, and floristic composition, without a homogeneous pattern.

Lopez-Gonzalez *et al.* [114] found remains of the diplocentrid scorpion *Diplocentrus peloncillensis* Francke, 1975, in seven scats (= droppings) from black bears in the Sierra de San Luis, Sonora. Surprisingly, scorpions of various class sizes were present in the scats; and the frequency of scorpion remains in bear scats (3.8%) indicates that scorpions do not constitute a large proportion of the diet of that black bear population. Nonetheless, this is the first report of bears feeding on scorpions anywhere in the world.

Quijano-Ravell *et al.* [115] determined the life cycle of the fossorial caraboctonid scorpion *Hoffmannihadrurus gertschi* (Soleglad, 1976) from field observations and monthly sampling during one year in deciduous scrub forest in central Guerrero. This species has seven stages (= instars) during its life history and requires at least four years to attain sexual maturity. Mating occurs at the end of the rainy season (July–October), and parturition occurs the following spring (April–May); thus, the gestation period lasts seven to eight months.

The same authors [116] analyzed the density, spatial distribution and biomass of *H. gertschi* at the same location as above. Densities varied from 0.35 scorpions per square meter down to 0.23/m^2^; an intermediate value among others reported for North American scorpions. Their spatial distribution is clumped (as opposed to uniform or scattered), evidenced by the young scorpions not travelling very far from the mother’s burrow. Finally, the estimated biomass of 6.2 kg of scorpions per hectare is comparatively similar to studies made in Australia and California with other species of burrowing scorpions.

In another contribution [117], the burrows of *H. gertschi* were examined. A total of 41 burrows of differently aged scorpions were excavated and measured: burrow entrance width, burrow total depth and burrow total length were positively correlated with the carapace length of the scorpion inhabiting each burrow. Each time a young scorpion prepares to molt, it seals its burrow from the inside; after it molts, it opens the entrance and enlarges its burrow, as evidenced by fresh tumuli of dirt at the burrow entrance containing remains of prey and of the exuvium shed during the molt.

Finally, Quijano-Ravell & Ponce-Saavedra [118] analyzed litter size in *Centruroides ornatus* Pocock, 1902, and found that the average number of young on the back of females differs significantly from the average number of embryos carried by pregnant females. That reduction in litter size could be due to a number of causes, both before, during, and after parturition.

## Supplementary Materials

The following are available online at www.mdpi.com/2072-6651/8/1/2/s1.
