# Supplementary material for: Scorpions from Mexico: From Species Diversity to Venom Complexity
Source: Toxins (Basel). 2015 Dec 24;8(1):2. doi: 10.3390/toxins8010002 (PMC4728524; doi:10.3390/toxins8010002)
Supplement: Supplementary file 1 [file toxins-08-00002-s001.pdf]

Supplementary Material: Scorpions from Mexico: From Species Diversity to Venom Complexity

Scorpion families, genera and species recorded from the states of Mexico. Abbreviations: (AGS) Aguascalientes; (BC) Baja California; (BCS) Baja California Sur; (CAM) Campeche; (CHIS) Chiapas; (CHIH) Chihuahua; (COA) Coahuila; (COL) Colima; (DF) Distrito Federal; (DUR) Durango; (GTO) Guanajuato; (GRO) Guerrero; (HGO) Hidalgo; (JAL) Jalisco; (MEX) Estado de México; (MICH) Michoacán; (MOR) Morelos; (NAY) Nayarit; (NL) Nuevo León; (OAX) Oaxaca; (PUE) Puebla; (QRO) Querétaro; (QROO) Quintana Roo; (SLP) San Luis Potosí; (SIN) Sinaloa; (SON) Sonora; (TABS) Tabasco; (TAMS) Tamaulipas; (TLAX) Tlaxcala; (VER) Veracruz; (YUC) Yucatán; (ZAC) Zacatecas. Asterisk denotes families, genera and species endemic to Mexico. * Asterisk denotes families, genera and species endemic to Mexico.

1. Family Buthidae C.L. Koch, 1837

1.1. Genus Centruroides Marx, 1890

Centruroides baergi Hoffmann, 1932: OAX, PUE *
Centruroides balsasensis Ponce-Saavedra & Francke, 2004: GRO, MEX, MICH, MOR, PUE *
Centruroides bertholdii (Thorell, 1876): JAL, MICH *
Centruroides chamela Ponce-Saavedra & Francke, 2011: JAL *
Centruroides chamulaensis Hoffmann, 1932: CHIS *
Centruroides chiapanensis Hoffmann, 1932: CHIS, OAX *
Centruroides edwardsii (Gervais, 1843): GRO, SON
Centruroides elegans (Thorell, 1876): COL, GRO, JAL, MICH, NAY *
Centruroides exilicauda (Wood, 1863): BC, BCS
Centruroides flavopictus (Pocock, 1898): OAX, VER *
Centruroides franckei Santibáñez-López & Contreras-Félix, 2013: OAX *
Centruroides fulvipes (Pocock, 1898): OAX *
Centruroides gracilis (Latreille, 1804): CAM, CHIS, HGO, OAX, QRO, QROO, SLP, TABS, TAMS, VER, YUC
Centruroides hirsutipalpus Ponce-Saavedra & Francke, 2009: COL *
Centruroides hoffmanni Armas, 1996: CHIS, OAX *
Centruroides infamatus (C.L. Koch, 1844): AGS, COL, DUR, GTO, JAL, MICH, NAY, QRO, SIN, ZAC *
Centruroides limpidus (Karsch, 1879): GRO, MEX, MICH, MOR, OAX, PUE, QRO *
Centruroides margaritatus (Gervais, 1841): MOR
Centruroides mascota Ponce-Saavedra & Francke, 2011: JAL *
Centruroides meisei Hoffmann, 1939: GRO *
Centruroides meridionalis Hoffmann, 1932: CHIS *
Centruroides nigrescens (Pocock, 1898): GRO, MICH, OAX *
Centruroides nigrimanus (Pocock, 1898): OAX *
Centruroides nigrovariatus (Pocock, 1898): OAX *
Centruroides noxius Hoffmann, 1932: NAY, SIN *
Centruroides ochraceus (Pocock, 1898): CAM, QROO, VER, YUC *
Centruroides ornatus Pocock, 1902: GTO, JAL, MICH, NAY *
Centruroides orizaba Armas & Martín-Frías, 2003: PUE, VER *
Centruroides pallidiceps Pocock, 1902: SIN, SON *
Centruroides poncei Teruel *et al.*, 2015: OAX *
Centruroides rileyi Sissom, 1995: SLP, TAMS *
Centruroides rodolfoi Santibáñez-López & Contreras-Félix, 2013: GRO, OAX *
Centruroides schmidti Sissom, 1995: CAM, CHIS, QROO, VER
Centruroides sculpturatus Ewing, 1928: SON
Centruroides serrano Santibáñez-López & Ponce-Saavedra, 2009: OAX *
Centruroides sissomi Armas, 1996: QROO *

Centruroides suffusus (Pocock, 1902): DUR, GTO, NAY, SIN, ZAC *
Centruroides tapachulaensis Hoffmann, 1932: CHIS *
Centruroides tecomanus Hoffmann, 1932: COL, JAL, MICH *
Centruroides tuxlta Armas, 1999: CHIS*
Centruroides villegasi Baldazo-Monsivais *et al.*, 2013: GRO *
Centruroides vittatus (Say, 1821): CHIH, COA, DUR, NL, TAMS, ZAC

*1.2. Genus Chaneke Francke et al., 2014 **

Chaneke aliciae (Armas & Martín-Frías, 1998): OAX
Chaneke fogoso Francke *et al.*, 2014: GRO

2. Family Caraboctonidae Kraepelin, 1905

2.1. Genus Anuroctonus Pocock, 1893

Anuroctonus pococki Soleglad & Fet, 2004: BC

2.2. Genus Hadrurus Thorell, 1876

Hadrurus anzaborrego Soleglad *et al.*, 2011: BC
Hadrurus arizonensis Ewing, 1928: BC, SON
Hadrurus concolorous Stahnke, 1976: BC, BCS *
Hadrurus hirsutus (Wood, 1863): BCS *
Hadrurus obscurus Williams, 1970: BC
Hadurus pinteri Stahnke, 1969: BC, BCS *

2.3. Genus Hoffmannihadrurus Soleglad & Fet, 2004

Hoffmannihadrurus aztecus (Pocock, 1902): OAX, PUE *
Hoffmannihadrurus gertschi (Soleglad, 1976): GRO, MOR *

3. Family Chactidae Pocock, 1893

Genus *Nullibrotheas* Williams, 1974 *

Nullibrotheas allenii (Wood, 1863): BC

4. Family Diplocentridae Karsch, 1880

*4.1. Genus Bioculus Stahnke, 1968 **

Bioculus caboensis (Stahnke, 1968): BCS
Bioculus cerralvensis Stahnke, 1968: BCS
Bioculus comondae Stahnke, 1968: BCS
Bioculus cruzensis Stahnke, 1968: BCS
Bioculus parvulus Martín-Frías, 2004: GRO

4.2. Genus Diplocentrus Peters, 1861

Diplocentrus actun Armas & Palacios-Vargas, 2002: YUC *
Diplocentrus anophthalmus Francke, 1977: YUC *
Diplocentrus bellator Teruel, 2003: GRO *
Diplocentrus bereai Armas & Martín-Frías, 2004: VER *
Diplocentrus bicolor Contreras-Félix & Santibáñez-López, 2011: JAL, ZAC *
Diplocentrus chiapasensis Beutelspacher & Armas, 1998: CHIS *
Diplocentrus chol Francke, 2007: CHIS *
Diplocentrus churumuco Francke & Ponce-Saavedra, 2003: MICH *
Diplocentrus colwelli Sissom, 1986: NL *

Diplocentrus coylei Fritts & Sissom, 1996: GRO, MEX, MOR *
Diplocentrus cozumel Beutelspacher & Armas, 1998: QROO *
Diplocentrus cueva Francke, 1978: OAX *
Diplocentrus diablo Stockwell & Nilsson, 1987: TAMS
Diplocentrus ferrugineus Fritts & Sissom, 1996: NL *
Diplocentrus formosus Armas & Martín-Frías, 2003: OAX *
Diplocentrus franckei Santibáñez-López, 2014: OAX *
Diplocentrus gertschi Sissom & Walker, 1992: JAL, NAY, SIN, SON *
Diplocentrus gladiator Beutelspacher & Trujillo, 1999: CHIS *
Diplocentrus hoffmanni Francke, 1977: OAX *
Diplocentrus karschi Francke & Sissom, 1985: OAX *
Diplocentrus jaca Armas & Martín-Frías, 2000: OAX *
Diplocentrus keyserlingii Karsch, 1880: OAX *
Diplocentrus kraepelini Santibáñez-López *et al.*, 2013: OAX *
Diplocentrus lindo Stockwell & Baldwin, 2001: COA, NL, TAMS
Diplocentrus longimanus Santibáñez-López *et al.*, 2011: MOR, PUE *
Diplocentrus luisae Guijosa, 1973: CAM, QROO *
Diplocentrus majahuensis Baldazo-Monsivais, 2003: GRO *
Diplocentrus melici Armas, Martín-Frías & Berea, 2004: VER *
Diplocentrus mexicanus Peters, 1861: OAX *
Diplocentrus mitchelli Francke, 1977: CAM *
Diplocentrus mitlae Francke, 1977: OAX *
Diplocentrus montecristo Armas & Martín-Frías, 2000: CHIS *
Diplocentrus ochoterenai Hoffmann, 1931: OAX *
Diplocentrus peloncillensis Francke, 1975: CHIH, SON
Diplocentrus perezi Sissom, 1991: VER *
Diplocentrus rectimanus Pocock, 1898: OAX *
Diplocentrus reddelli Francke, 1977: CAM, QROO, YUC *
Diplocentrus roo Armas & Martín-Frías, 2005: QROO *
Diplocentrus sagittipalpus Santibáñez-López *et al.*, 2013: OAX *
Diplocentrus silanesi Armas & Martín-Frías, 2000: MEX, MICH *
Diplocentrus sinaan Armas & Martín-Frías, 2000: CHIS *
Diplocentrus sissomi Santibáñez-López *et al.*, 2013: OAX *
Diplocentrus spitzeri Stahnke, 1970: SON
Diplocentrus steeleae Stockwell, 1986: CHIS *
Diplocentrus taibeli (Caporiacco, 1938): CAM, QROO, YUC
Diplocentrus tehuacanus Hoffmann, 1931: GRO, MOR, OAX, PUE *
Diplocentrus tehuano Francke, 1977: OAX *
Diplocentrus tenango Santibáñez-López & Francke, 2008: OAX *
Diplocentrus whitei (Gervais, 1844): CHIH, COA, NL, TAMS
Diplocentrus williamsi Sissom & Wheeler, 1995: SON *
Diplocentrus zacatecanus Hoffmann, 1931: AGS, DUR, GTO, HGO, MEX, QRO, SLP, ZAC *

*4.3. Genus Kolotl Santibáñez-López et al., 2014 **

Kolotl magnus (Beutelspacher & López-Forment, 1991): GRO
Kolotl poncei (Francke & Quijano-Ravell, 2009): MICH

5. Family Euscorpiidae Laurie, 1896

*5.1. Genus Megacormus Karsch, 1881 **

Megacormus gertschi Díaz-Najera, 1966: HGO, PUE, QRO, SLP, TAMS, VER
Megacormus granosus (Gervais, 1844): VER
Megacormus grubbsi Sissom, 1994: OAX

Megacormus segmentatus (Pocock, 1900): VER

5.2. Genus Plesiochactas Pocock, 1900

Plesiochactas dilutus Pocock, 1900: OAX, VER *
Plesiochactas mitchelli Soleglad, 1976: CHIS

*5.3. Genus Troglocormus Francke, 1981 **

Troglocormus ciego Francke, 1981: SLP
Troglocormus willis Francke, 1981: TAMS

6. Family Superstitiionidae Stahnke, 1940

Genus Superstitionia Stahnke, 1940

Superstitionia donensis Stahnke, 1940: BC, BCS, SON

7. Family Typhlochactidae Mitchell, 1971 *

7.1. Genus Alacran Francke, 1982

Alacran chamuco Francke, 2009: OAX
Alacran tartarus Francke, 1982: OAX
Alacran triquimera Santibáñez-López *et al.*, 2014: PUE

7.2. Genus Sotanochactas Francke, 1982

Sotanochactas ellioti (Mitchell, 1971): SLP

7.3. Genus Stygochactas Vignoli & Prendini, 2009

Stygochactas granulosus (Sissom & Cokendolpher, 1998): VER

7.4. Genus Typhlochactas Mitchell, 1971

Typhlochactas cavicola Francke, 1986: TAMS
Typhlochactas mitchelli Sissom, 1988: OAX
Typhlochactas reddelli Mitchell, 1968: VER
Typhlochactas rhodesi Mitchell, 1968: TAMS
Typhlochactas sissomi Francke *et al.*, 2009: QRO
Typhlochactas sylvestris Mitchell & Peck, 1977: OAX

8. Family Vaejovidae Thorell, 1876

*8.1. Genus Balsateres González-Santillán & Prendini, 2013 **

Balsateres cisnerosi (Ponce-Saavedra & Sissom, 2004): MICH

8.2. Genus Chihuahuanus González-Santillán & Prendini, 2013

Chihuahuanus bilineatus (Pocock, 1898): AGS, COA, GTO, NL, SLP, TAMS *
Chihuahuanus cazieri (Williams, 1968): COA, NL *
Chihuahuanus coahuilae (Williams, 1968): CHIH, COA
Chihuahuanus crassimanus (Pocock, 1898): DUR, NL
Chihuahuanus glabrimanus (Sissom & Hendrixson, 2005): COA, NL *
Chihuahuanus globosus (Borelli, 1915): COA, DUR
Chihuahuanus kovariki (Soleglad & Fet, 2008): DUR *
Chihuahuanus russelli (Williams, 1971): SON

*8.3. Genus Franckeus Soleglad & Fet, 2005 **

Franckeus kochi (Sissom, 1991): HGO, MEX, QRO
Franckeus nitidulus (C.L. Koch, 1843): HGO, MEX, QRO
Franckeus peninsularis (Williams, 1980): BCS
Franckeus platnicki (Sissom, 1991): SLP, TAMS
Franckeus rubrimanus (Sissom, 1991): NL

8.4. Genus Gertschius Graham & Soleglad, 2007

Gertschius agilis (Sissom & Stockwell, 1991): SON
Gertschius crassicorpus Graham & Soleglad, 2007: SON *

8.5. Genus Kochius Soleglad & Fet, 2008

Kochius barbatus (Williams, 1971): BCS *
Kochius bruneus (Williams, 1970): BCS *
Kochius cerralvensis (Williams, 1971): BCS *
Kochius hirsuticauda (Banks, 1910): BC
Kochius insularis Williams, 1971: BCS *
Kochius magdalensis Williams, 1971: BCS *
Kochius punctipalpi (Wood, 1863): BCS *
Kochius sonorae Williams, 1971: SON *
Kochius villosus Williams, 1970: BC, BCS *

*8.6. Genus Konetontli González-Santillán & Prendini, 2013 **

Konetontli acapulco (Armas & Martín-Frías, 2001): GRO
Konetontli chamelaensis (Williams, 1986): JAL
Konetontli ignes González-Santillán & Prendini, 2015: GRO
Konetontli ilitchi González-Santillán & Prendini, 2015: COL
Konetontli juxtlahuaca González-Santillán & Prendini, 2015: GRO
Konetontli kuarapu (Francke & Ponce-Saavedra, 2005): MICH
Konetontli migrus González-Santillán & Prendini, 2015: GRO
Konetontli nayarit (Armas & Martín-Frías, 2001): NAY
Konetontli pattersoni (Williams, 1980): BCS
Konetontli zihuatanejensis (Baldazo-Monsivaiz, 2003): GRO

*8.7. Genus Kuarapu Francke & Ponce-Saavedra, 2010 **

Kuarapu purhepecha Francke & Ponce-Saavedra, 2010: MICH

8.8. Genus Maaykuyak González-Santillán & Prendini 2013

Maaykuyak vittatus (Williams, 1970): BCS *
Maaykuyak waueri (Gertsch & Soleglad, 1972): CHIH, COA, DUR, NL

*8.9. Genus Mesomexovis González-Santillán & Prendini, 2013 **

Mesomexovis atenango (Francke & González-Santillán, 2006): GRO
Mesomexovis oaxaca (Santibáñez-López & Sissom, 2009): CHIS, OAX
Mesomexovis occidentalis (Hoffmann, 1931): GRO, MICH, OAX
Mesomexovis punctatus (Karsch, 1879): GTO, HGO, MEX, QRO, TLAX
Mesomexovis spadix (Hoffmann, 1931): AGS, JAL, ZAC
Mesomexovis subcristatus (Pocock, 1898): GRO, MOR, OAX, PUE, VER
Mesomexovis variegatus (Pocock, 1898): GRO, MEX, MICH, MOR, PUE

8.10. Genus Paravaejovis Williams, 1980

Paravaejovis confusus (Stahnke, 1940): BC, SON
Paravaejovis diazi (Williams, 1970): BCS *
Paravaejovis eusthenura (Wood, 1863): BCS *
Paravaejovis galbus (Williams, 1970): BCS *
Paravaejovis gravicaudus (Williams, 1970): BC, BCS *
Paravaejovis hoffmanni (Williams, 1970): BC, BCS *
Paravaejovis pumilis (Williams, 1970): BCS *
Paravaejovis puritanus (Gertsch, 1958): BC, BCS
Paravaejovis schwenkmeyeri (Williams, 1970): BC, BCS *
Paravaejovis spinigerus (Wood, 1863): BC, SON
Paravaejovis waeringi (Williams, 1970): BC

8.11. Genus Paruroctonus Werner, 1934

Paruroctonus arnaudi Williams, 1972: BC *
Paruroctonus baergi (Williams & Hadley, 1967): SON
Paruroctonus bajae Williams, 1972: BC *
Paruroctonus borregoensis Williams, 1972: BC, SON
Paruroctonus coahuilanus Haradon, 1985: COA *
Paruroctonus gracilior (Hoffmann, 1931): AGS, CHIH, COA
Paruroctonus luteolus (Gertsch & Soleglad, 1966): BC
Paruroctonus nitidus Haradon, 1984: BC *
Paruroctonus pseudopumilis (Williams, 1970): BCS *
Paruroctonus silvestrii (Borelli, 1909): BC
Paruroctonus surensis Williams & Hardon, 1980: BCS *
Paruroctonus stahnkei (Gertsch & Soleglad, 1966): SON
Paruroctonus utahensis (Williams, 1968): CHIH
Paruroctonus ventosus Williams, 1972: BC *
Paruroctonus xanthus (Gertsch & Soleglad, 1966): SON

8.12. Genus Pseudouroctonus Stahnke, 1974

Pseudouroctonus andreas (Gerstch & Soleglad, 1972): BC
Pseudouroctonus apacheanus (Gerstch & Soleglad, 1972): CHIH, SON
Pseudouroctonus cazieri (Gerstch & Soleglad, 1972): BC *
Pseudouroctonus chicano (Gerstch & Soleglad, 1972): CHIH *
Pseudouroctonus lindsayi (Gerstch & Soleglad, 1972): BCS *
Pseudouroctonus reddelli (Gerstch & Soleglad, 1972): COA, NL, TAMS
Pseudouroctonus rufulus (Gerstch & Soleglad, 1972): BC *
Pseudouroctonus savvasi Francke, 2009: COA *
Pseudouroctonus sprousei Francke & Savary, 2006: COA *

8.13. Genus Serradigitus Stahnke, 1974

Serradigitus adcocki (Williams, 1980): BCS *
Serradigitus armadentis (Williams, 1980): BCS *
Serradigitus baueri (Gertsch, 1958): BC *
Serradigitus bechteli (Williams, 1980): BCS *
Serradigitus calidus (Soleglad, 1974): COA *
Serradigitus dwyeri (Williams, 1980): BCS *
Serradigitus gertschi (Williams, 1968): BC, BCS
Serradigitus gigantaensis (Williams, 1980): BCS *
Serradigitus haradoni (Williams, 1980): BCS *

Serradigitus hearnei (Williams, 1980): BC, BCS, SON *
Serradigitus littoralis (Williams, 1980): BC, BCS *
Serradigitus minutis (Williams, 1970): BCS *
Serradigitus pacificus (Williams, 1980): BC *
Serradigitus yaqui Sissom & Stockwell, 1991: SON *

8.14. Genus Smeringurus Haradon, 1983

Smeringurus grandis (Williams, 1970): BC *
Smeringurus mesaensis (Stahnke, 1957): BC, SON

8.15. Genus Stahnkeus Soleglad & Fet, 2006

Stahnkeus allredi (Sissom & Stockwell, 1991): SON
Stahnkeus harbisoni (Williams, 1970): BC
Stahnkeus polisi (Sissom & Stockwell, 1991): SON *
Stahnkeus subtilimanus (Soleglad, 1972): SON

*8.16. Genus Syntropis Kraepelin, 1900 **

Syntropis aalbui Lowe *et al.*, 2007: BC
Syntropis macrura Kraepelin, 1900: BCS
Syntropis williamsi Soleglad *et al.*, 2007: BCS

*8.17. Genus Thorellius Soleglad & Fet, 2008 **

Thorellius atrox (Hoffmann, 1931): AGS, COL, JAL
Thorellius cristimanus (Pocock, 1898): AGS, GTO, JAL, MEX, MICH, ZAC
Thorellius intrepidus (Thorell, 1876): COL, JAL, MICH

8.18. Genus Uroctonites Williams & Savary, 1991

Uroctonites huachuca (Gertsch & Soleglad, 1972): SON

8.19. Genus Vaejovis C.L. Koch, 1836

Vaejovis bandido Graham *et al.*, 2012: SON *
Vaejovis chiapas Sissom, 1989: CHIS *
Vaejovis coalocoman Contreras-Félix & Francke, 2014: MICH *
Vaejovis curvidigitus Sissom, 1991: GRO, MEX, MOR, OAX, PUE *
Vaejovis darwini Santibáñez-López & Francke, 2010: OAX *
Vaejovis davidi Soleglad & Fet, 2005: PUE *
Vaejovis decipiens Hoffmann, 1931: CHIH, SON *
Vaejovis dugesi Pocock, 1902: GTO *
Vaejovis dzahui Santibáñez-López & Francke, 2010: OAX *
Vaejovis franckei Sissom, 1989: OAX *
Vaejovis gracilis Gertsch & Soleglad, 1972: VER *
Vaejovis granulatus Pocock, 1898: DF, MEX, MICH, MOR *
Vaejovis intermedius Borelli, 1915: CHIH, COA, DUR, NL
Vaejovis janssi Williams, 1980: BCS *
Vaejovis maculosus Sissom, 1989: PUE *
Vaejovis mauryi Capes, 2001: SON *
Vaejovis mexicanus C.L. Koch, 1836: DF, MEX, MOR *
Vaejovis minckleyi Williams, 1968: COA *
Vaejovis mitchelli Sissom, 1991: QRO, SLP *
Vaejovis montanus Graham & Bryson, 2010: SON
Vaejovis monticola Sissom, 1989: COL, JAL *

Vaejovis morelia Miranda-López *et al.*, 2012: MICH *
Vaejovis nigrescens Pocock, 1898: AGS, GTO, JAL, MEX, MICH, QRO, ZAC *
Vaejovis nigrofemoratus Hendrixson & Sissom, 2001: OAX *
Vaejovis norteno Sissom & González-Santillán, 2004: COA, NL *
Vaejovis ocotensis Zárate-Gálvez & Francke, 2009: CHIS *
Vaejovis pequeno Hendrixson, 2001: SON *
Vaejovis pococki Sissom, 1991: GTO, QRO, SLP *
Vaejovis prendinii Santibáñez-López & Francke, 2010: OAX *
Vaejovis pusillus Pocock, 1898: GRO, MEX, MICH *
Vaejovis rossmani Sissom, 1989: NL, TAMS *
Vaejovis setosus Sissom, 1989: OAX *
Vaejovis smithi Pocock, 1902: GRO, MOR *
Vaejovis solegladi Sissom, 1991: OAX, PUE *
Vaejovis sprousei Sissom, 1990: NL, TAMS *
Vaejovis tenamaztlei Contreras-Félix *et al.*, 2015: AGS *
Vaejovis tesselatus Hendrixson & Sissom, 2001: SLP *
Vaejovis trespicos Zárate-Gálvez & Francke, 2009: CHIS *
Vaejovis vaquero Gertsch & Soleglad, 1972 : CHIH *
Vaejovis zapoteca Santibáñez-López & Francke, 2010: OAX *

*8.20. Genus Vizcaino González-Santillán & Prendini, 2013 **

Vizcaino viscainensis (Williams, 1970): BC, BCS

*8.21. Genus Vejovoidus Stahnke, 1974 **

Vejovoidus longiunguis (Williams, 1969): BCS
